# Oscillation of certain higher-order neutral partial functional differential equations

**DOI:** 10.1186/s40064-016-2111-y

**Published:** 2016-04-14

**Authors:** Wei Nian Li, Weihong Sheng

**Affiliations:** Department of Mathematics, Binzhou University, Binzhou, Shandong 256603 People’s Republic of China

**Keywords:** Oscillation, Partial functional differential equation, Robin boundary condition, 35B05, 35R10

## Abstract

In this paper, we study the oscillation of certain higher-order neutral partial functional differential equations with the Robin boundary conditions. Some oscillation criteria are established. Two examples are given to illustrate the main results in the end of this paper.

## Background

It is well known that the theory of partial functional differential equations can be applied to many fields, such as population dynamics, cellular biology, meteorology, viscoelasticity, engineering, control theory, physics and chemistry (Wu 1996). In the monograph, Wu (1996) provided some fundamental theories and applications of partial functional differential equations.

The oscillation theory as a part of the qualitative theory of partial functional differential equations has been developed in the past few years. Many researchers have established some oscillation results for partial functional differential equations. For example, see the monograph (Yoshida 2008) and the papers (Bainov et al. 1996; Fu and Zhuang 1995; Li and Cui 1999; Li 2000; Li and Cui 2001; Ouyang et al. 2005; Gao and Luo 2008; Li and Han 2006; Wang et al. 2010). We especially note that the monograph (Yoshida 2008) contained large material on oscillation theory for partial differential equations.

Li and Cui (2001) studied the oscillation of even order partial functional differential equations

E1 $$\begin{aligned} & \frac{\partial ^{n}}{\partial t^{n}}[u(x,t)+\mu (t)u(x,t-\rho )]=a(t)\Delta u(x,t) +\sum _{k=1}^{s}a_k(t)\Delta u(x,\rho _k(t))\\ & \quad -q(x,t)u(x,t) - \int _{a}^{b}p(x,t,\xi )u(x,g(t,\xi )){\mathrm{d}}\sigma (\xi ), \quad (x,t)\in \Omega \times [0,\infty )\equiv G, \end{aligned}$$

where $n\ge 2$ is an even integer, with the two kinds of boundary conditions:

B1 $$\begin{aligned} \frac{\partial u(x,t)}{\partial N}+\nu (x,t)u(x,t)=0, \quad (x,t)\in \partial \Omega \times [0,\infty ), \end{aligned}$$

and

B2 $$\begin{aligned} u(x,t)=0, \quad (x,t)\in \partial \Omega \times [0,\infty ). \end{aligned}$$

Ouyang et al. (2005) established the oscillation of odd order partial functional differential equations

E2 $$\begin{aligned} & \frac{\partial ^{n}u(x,t)}{\partial t^{n}}-a(t)\Delta u(x,t) -\sum _{k=1}^{s}p_k(x,t)u(x,t-\sigma _k)-\sum _{j=1}^mq_j(x,t)u(x,t-\tau _j)\\ &\quad +\, h(t)f(u(x,t-r_1),\cdots ,u(x,t-r_{\ell }))=0, \quad (x,t)\in \Omega \times [0,\infty )\equiv G, \end{aligned}$$

where *n* is an odd integer and $s\le m$, with the boundary conditions (*B*1), (*B*2) and

B3 $$\begin{aligned} \frac{\partial u(x,t)}{\partial N}=0, \quad (x,t)\in \partial \Omega \times [0,\infty ).\qquad {} \end{aligned}$$

In this paper, we investigate the oscillation of the following higher-order neutral partial functional differential equations

1 $$\begin{aligned} & \frac{\partial ^{n}}{\partial t^{n}}[u(x,t)+\mu (t)u(x,t-\tau )]=a(t)\Delta u(x,t) +\sum _{k=1}^{s}a_k(t)\Delta u(x,\rho _k(t))\\ &\quad -\, \int _{a}^{b}p(t,\xi )u(x,g(t,\xi )){\mathrm{d}}\sigma (\xi ), \quad \ (x,t)\in \Omega \times [0,\infty )\equiv G, \end{aligned}$$

with the Robin boundary condition

2 $$\begin{aligned} \alpha (x)\frac{\partial u(x,t)}{\partial N}+\beta (x)u(x,t)=0, \quad (x,t)\in \partial \Omega \times [0,\infty ), \end{aligned}$$

where $$n\ge 2$$ is an even integer, $$\Omega$$ is a bounded domain in $${\mathbb{R}}^{M}$$ with a piecewise smooth boundary $$\partial \Omega$$, and $$\Delta$$ is the Laplacian in the Euclidean *M*-space $${\mathbb{R}}^M$$, $$\alpha ,\beta \in C(\partial \Omega ,[0,\infty )),$$$$\alpha ^2(x)+\beta ^2(x)\ne 0$$, and *N* is the unite exterior normal vector to $$\partial \Omega$$.

Throughout this paper, we always suppose that the following conditions hold:

1. $$\mu \in C^{n}([0,\infty );[0,\infty )), 0\le \mu (t)\le 1,\tau =$$ const.$${>}0;$$
2. $$a,a_k\in C([0,\infty );[0,\infty )),\rho _k\in C([0,\infty );[0,\infty )),\rho _k(t)\le t,$$$$\lim _{t\rightarrow +\infty }\rho _k(t)=+\infty , \ k\in I_s=\{1,2,\ldots ,s\};$$
3. $$p\in C([0,\infty )\times [a,b];[0,\infty )),$$$$g\in C([0,\infty )\times [a,b];[0,\infty )),$$$$g(t,\xi )\le t,\xi \in [a,b],$$$$g(t,\xi )$$ is a nondecreasing function with respect to *t* and $$\xi$$, respectively, and $$\lim _{t\rightarrow +\infty }\inf _{\xi \in [a,b]}\{g(t,\xi )\}=+\infty ;$$
4. $\sigma \in ([a,b];{\mathbb{R}})$ and $\sigma (\xi )$ is nondecreasing in $\xi$, the integral in (1) is Stieltjes integral.

As it is customary, the solution $u(x,t)\in C^n(G)\bigcap C^1(\overline{G})$ of the problem (1), (2) is said to be oscillatory in the domain $G\equiv \Omega \times [0,\infty )$ if for any positive number $\mu$ there exists a point $(x_0,t_0)\in \Omega \times [\mu ,\infty )$ such that the equality $u(x_0,t_0)=0$ holds.

To the best of our knowledge, no result is known regarding the oscillatory behavior of higher-order partial functional differential equations with the Robin boundary condition (2) up to now.

The paper is organized as follows. In “Main results” section, we establish some results for the oscillation of the problem (1), (2). In “Examples” section, we construct two examples to illustrate our main results.

## Main results

In this section, we establish the oscillation criteria of the problem (1), (2). First, we introduce the following lemma which is very useful for establishing our main results.

### **Lemma 1**

Ye and Li (1990). *Suppose that*$\lambda _0$*is the smallest eigenvalue of the problem*

3 $$\begin{aligned} \left\{ \begin{array}{lll}\Delta \varphi (x)+\lambda \varphi (x)=0, \quad in \ \Omega ,\\ \alpha (x) \frac{\partial \varphi (x)}{\partial N}+\beta (x)\varphi (x)=0, \quad on \ \partial \Omega \end{array} \right. \end{aligned}$$

*and*$$\varphi (x)$$*is the corresponding eigenfunction of*$$\lambda _0$$. *Then*$$\lambda _0=0, \varphi (x)=1$$*as*$$\beta (x)=0$$$$(x\in \Omega )$$*and*$$\lambda _0>0,\varphi (x)>0$$$$(x\in \Omega )$$*as*$$\beta (x)\not \equiv 0$$$$(x\in \partial \Omega ).$$

Next, we give our main results.

### **Theorem 2**

*If*$\beta (x)\not \equiv 0$*for*$x\in \partial \Omega$, *then the necessary and sufficient condition for all solutions of the problem* (1), (2) *to oscillate is that all solutions of the differential equation*

4 $$\begin{aligned} & [y(t)+\mu (t)y(t-\tau )]^{(n)}+\lambda _0a(t)y(t)+\lambda _0\sum _{k=1}^{s}a_{k}(t)y(\rho _k(t))\\ &\quad +\int _{a}^{b}p(t,\xi )y(g(t,\xi )){\mathrm{d}}\sigma (\xi )=0, \quad t\ge 0, \end{aligned}$$

*to oscillate, where*$\lambda _0$*is the smallest eigenvalue of* (3).

### *Proof*

(i) Sufficiency. Suppose to the contrary that there is a non-oscillatory solution *u*(*x*, *t*) of the problem (1), (2) which has no zero in $$\Omega \times [t_0,\infty )$$ for some $$t_0\ge 0$$. Without loss of generality we assume that $$u(x,t)>0, \ u(x,t-\tau )>0, \ u(x,\rho _k(t))>0,$$$$u(x,g(t,\xi ))>0$$, $$(x,t)\in \Omega \times [t_1,\infty ),k\in I_s.$$

Multiplying both sides of (1) by $\varphi (x)$ and integrating with respect to *x* over the domain $\Omega$, we have

5 $$\begin{aligned} &\frac{\mathrm{d}^{n}}{\mathrm{d}t^{n}}\bigg[\int _{\Omega }u(x,t)\varphi (x){\mathrm{d}}x+\mu (t) \int _{\Omega }u(x,t-\tau )\varphi (x){\mathrm{d}}x\bigg]\\ &\quad =a(t) \int _{\Omega }\Delta u(x,t)\varphi (x){\mathrm{d}}x + \sum _{k=1}^{s}a_{k}(t)\int _{\Omega }\Delta u(x,\rho _k(t))\varphi (x){\mathrm{d}}x\\ &\qquad -\, \int _{\Omega }\int _{a}^{b}p(t,\xi )u(x,g(t,\xi ))\varphi (x){\mathrm{d}}\sigma (\xi ){\mathrm{d}}x, \quad t\ge t_1. \end{aligned}$$

From Green’s formula and boundary condition (2), it follows that

$$\begin{aligned} & \int _{\Omega }\Delta u(x,t)\varphi (x){\mathrm{d}}x\\ &\quad = \int _{\partial \Omega }\bigg (\varphi (x)\frac{\partial u(x,t)}{\partial N}-u(x,t)\frac{\partial \varphi (x)}{\partial N}\bigg ){\mathrm{d}}S+\int _{\Omega }u(x,t)\Delta \varphi (x){\mathrm{d}}x\\ &\quad = \int _{\partial \Omega }\bigg (\varphi (x)\frac{\partial u(x,t)}{\partial N}-u(x,t)\frac{\partial \varphi (x)}{\partial N}\bigg ){\mathrm{d}}S-\lambda _0\int _{\Omega }u(x,t)\varphi (x){\mathrm{d}}x, \quad t\ge t_1, \end{aligned}$$

where ${\mathrm{d}}S$ is the surface element on $\partial \Omega$.

If $\alpha (x)\equiv 0, x\in \partial \Omega ,$ then from (2) we have

$$\begin{aligned} \beta (x)\not \equiv 0, \ \ u(x,t)=0, \quad \ (x,t)\in \partial \Omega \times [0,\infty ).\end{aligned}$$

Hence, we obtain

$$\begin{aligned} \int _{\partial \Omega }\bigg (\varphi (x)\frac{\partial u(x,t)}{\partial N}-u(x,t)\frac{\partial \varphi (x)}{\partial N}\bigg ){\mathrm{d}}S\equiv 0, \quad t\ge t_1. \end{aligned}$$

If $\alpha (x)\not \equiv 0, x\in \partial \Omega .$ Noting that $\partial \Omega$ is piecewise smooth, $\alpha , \ \beta \in C(\partial \Omega ,[0,\infty )), \alpha ^2(x)+\beta ^2(x)\ne 0$, without loss of generality, we can assume that $\alpha (x)>0, \ x\in \partial \Omega .$ Then by (2) and (3) we have

$$\begin{aligned} &\int _{\partial \Omega }\bigg (\varphi (x)\frac{\partial u(x,t)}{\partial N}-u(x,t)\frac{\partial \varphi (x)}{\partial N}\bigg ){\mathrm{d}}S\\ & \quad = \int _{\partial \Omega }\bigg (-\varphi (x)\frac{\beta (x)}{\alpha (x)}u(x,t)+u(x,t)\frac{\beta (x)}{\alpha (x)} \varphi (x)\bigg ){\mathrm{d}}S=0, \quad t\ge t_1, \end{aligned}$$

Therefore, using Lemma 1, we obtain

6 $$\begin{aligned} \int _{\Omega }\Delta u(x,t)\varphi (x){\mathrm{d}}x=-\lambda _0\int _{\Omega }u(x,t)\varphi (x){\mathrm{d}}x, \quad t\ge t_1. \end{aligned}$$

Similarly, we have

7 $$\begin{aligned} \int _{\Omega }\Delta u(x,\rho _k(t))\varphi (x){\mathrm{d}}x=-\lambda _0\int _{\Omega }u(x,\rho _k(t))\varphi (x){\mathrm{d}}x, \quad t\ge t_1, \ k\in I_s. \end{aligned}$$

It is easy to see that

8 $$\begin{aligned} & \int _{\Omega }\int _{a}^{b}p(t,\xi )u(x,g(t,\xi ))\varphi (x){\mathrm{d}}\sigma (\xi ){\mathrm{d}}x \\ & \quad = \int _{a}^{b}p(t,\xi )\int _{\Omega }u(x,g(t,\xi ))\varphi (x){\mathrm{d}}x{\mathrm{d}}\sigma (\xi ), \quad t\ge t_1. \end{aligned}$$

Set

$$\begin{aligned} V(t)=\int _{\Omega }u(x,t)\varphi (x){\mathrm{d}}x, \ t\ge t_1. \end{aligned}$$

Combining (5)–(8) we have

9 $$\begin{aligned} & [V(t)+\mu (t)V(t-\tau )]^{(n)}+\lambda _0a(t)V(t)+\lambda _0\sum _{k=1}^{s}a_{k}(t)V(\rho _k(t))\\ & \quad+\int _{a}^{b}p(t,\xi )V(g(t,\xi )){\mathrm{d}}\sigma (\xi )=0, \quad t\ge t_1. \end{aligned}$$

Obviously, it follows from (9) that *V*(*t*) is a positive solution of Eq. (4), which contradicts the fact that all solutions of Eq. (4) are oscillatory.

(ii) Necessity. Suppose that Eq. (4) has a non-oscillatory solution $\widetilde{V}(t)>0$. Without loss of generality we assume $\widetilde{V}(t)>0$ for $t\ge t_{*}\ge 0$, where $t_{*}$ is some large number. From (4), we have

10 $$\begin{aligned} & [\widetilde{V}(t)+\mu (t)\widetilde{V}(t-\tau )]^{(n)}+\lambda _0a(t)\widetilde{V}(t)+\lambda _0\sum _{k=1}^{s}a_{k}(t)\widetilde{V}(\rho _k(t))\\ & \quad + \int _{a}^{b}p(t,\xi )\widetilde{V}(g(t,\xi )){\mathrm{d}}\sigma (\xi )=0, \quad t\ge t_{*}. \end{aligned}$$

Multiplying both sides of (10) by $\varphi (x)$, we obtain

11 $$\begin{aligned} & \frac{\partial ^n}{\partial t^n}\bigg [\widetilde{V}(t)\varphi (x)+\mu (t)\widetilde{V}(t-\tau )\varphi (x)\bigg ]\\ & \quad +\lambda _0a(t)\widetilde{V}(t)\varphi (x) +\lambda _0 \sum _{k=1}^{s}a_{k}(t)\widetilde{V}(\rho _k(t))\varphi (x)\\ & \quad + \int _{a}^{b}p(t,\xi )\widetilde{V}(g(t,\xi ))\varphi (x){\mathrm{d}}\sigma (\xi )=0, \quad t\ge t_*, \quad x\in \Omega . \end{aligned}$$

Let $\widetilde{u}(x,t)=\widetilde{V}(t)\varphi (x), \quad (x,t)\in \Omega \times [0,\infty )$. By Lemma 1, we have $\Delta \varphi (x)=-\lambda _0\varphi (x), \quad x\in \Omega$. Then (11) implies

12 $$\begin{aligned} & \frac{\partial ^{n}}{\partial t^n}\bigg [\widetilde{u}(x,t)+\mu (t)\widetilde{u}(x,t-\tau )\bigg ]= a(t)\Delta \widetilde{u}(x,t)+\sum _{k=1}^{s}a_{k}(t)\Delta \widetilde{u}(x,\rho _k(t))\\ & \quad -\int _{a}^{b}p(t,\xi )\widetilde{u}(x,g(t,\xi )){\mathrm{d}}\sigma (\xi ), \quad t\ge t_*, \ x\in \Omega , \end{aligned}$$

which shows that $\widetilde{u}(x,t)=\widetilde{V}(t)\varphi (x), \ (x,t)\in \Omega \times [t_*,\infty ),$ satisfies (1).

From Lemma 1, we get

$$\begin{aligned} \alpha (x) \frac{\partial \varphi (x)}{\partial N}+\beta (x)\varphi (x)=0, \quad x\in \partial \Omega , \end{aligned}$$

which implies

13 $$\begin{aligned} \alpha (x) \frac{\partial \widetilde{u}(x,t)}{\partial N}+\beta (x)\widetilde{u}(x,t)=0, \quad (x,t)\in \partial \Omega \times [0,\infty ). \end{aligned}$$

Hence $\widetilde{u}(x,t)=\widetilde{V}(t)\varphi (x)>0$ is a non-oscillatory solution of the problem (1), (2), which is a contradiction. The proof is complete. $\square$

### *Remark 3*

Theorem 2 shows that the oscillation of problem (1), (2) is equivalent to the oscillation of the differential equation (4).

### **Theorem 4**

*If*$\beta (x)\not \equiv 0$*for*$x\in \partial \Omega$, *and the neutral differential inequality*

14 $$\begin{aligned}{}[y(t)+\mu (t)y(t-\tau )]^{(n)}+\int _{a}^{b}p(t,\xi )y(g(t,\xi )){\mathrm{d}}\sigma (\xi )\le 0, \quad t\ge 0, \end{aligned}$$

*has no eventually positive solutions, then every solution of the problem* (1), (2) *is oscillatory inG*.

### *Proof*

Suppose to the contrary that there is a non-oscillatory solution *u*(*x*, *t*) of the problem (1), (2) which has no zero in $$\Omega \times [t_0,\infty )$$ for some $$t_0\ge 0$$. Without loss of generality we assume that $$u(x,t)>0, \ u(x,t-\tau )>0, \ u(x,\rho _k(t))>0,$$$$u(x,g(t,\xi ))>0$$, $$(x,t)\in \Omega \times [t_1,\infty ),k\in I_s.$$ As in the proof of Theorem 2, we obtain Eq. (9). By Lemma 1, from (9) we have

15 $$\begin{aligned} & [V(t)+\mu (t)V(t-\tau )]^{(n)} +\int _{a}^{b}p(t,\xi )V(g(t,\xi )){\mathrm{d}}\sigma (\xi )\\ &\quad = -\lambda _0a(t)V(t)-\lambda _0\sum _{k=1}^{s}a_{k}(t)V(\rho _k(t))\\ &\quad \le 0, \quad t\ge t_1, \end{aligned}$$

which shows that $V(t)>0$ is a solution of the inequality (14). This is a contradiction. The proof of Theorem 4 is complete. $\square$

Using Theorems 1 and 2 in Li and Cui (2001), we can obtain the following two conclusions, respectively.

### **Theorem 5**

*Assume that*$\beta (x)\not \equiv 0$*for*$x\in \partial \Omega$. *If for*$t_0>0,$

16 $$\begin{aligned} \int _{t_0}^{+\infty }\int _{a}^{b}p(s,\xi )[1-\mu (g(s,\xi ))]{\mathrm{d}}\sigma (\xi ){\mathrm{d}}s=+\infty , \end{aligned}$$

*then every solution of the problem* (1), (2) *is oscillatory inG*.

### **Theorem 6**

*Assume that*$\beta (x)\not \equiv 0$*for*$x\in \partial \Omega$, $\mu (t)\equiv \mu$*is a positive constant,*$p(t,\xi )$*is periodic in t with period*$\rho$. *If for*$t_0>0,$

17 $$\begin{aligned}&g(t-c,\xi )=g(t,\xi )-c \quad \text{ for } \text{ any } \text{ number } c>0, \end{aligned}$$

18 $$\begin{aligned}&\int _{t_0}^{+\infty }\int _{a}^{b}p(s,\xi ){\mathrm{d}}\sigma (\xi ){\mathrm{d}}s=+\infty , \end{aligned}$$

*then every solution of the problem* (1), (2) *is oscillatory inG*.

## Examples

In this section, we give two examples to illustrate our main results.

### *Example 7*

Consider the partial functional differential equation

19 $$\begin{aligned} & \frac{\partial ^6}{\partial t^6}\bigg [u(x,t)+\frac{1}{5}u(x,t-\pi )\bigg ]=3\Delta u(x,t)+ \frac{11}{5}\Delta u\left( x,t-\frac{3\pi }{2}\right) \\ & \quad -\int _{-\pi }^{-\pi /2}\frac{11}{5}u(x,t+\xi ){\mathrm{d}}\xi , \quad (x,t)\in (0,\pi )\times [0,\infty ), \end{aligned}$$

with the boundary condition

20 $$\begin{aligned} u(0,t)=u(\pi ,t)=0, \quad t\ge 0. \end{aligned}$$

Here $n=6,\mu (t)=\frac{1}{5},\tau =\pi ,a(t)=3,a_1(t)=\frac{11}{5} ,\rho _1(t)=t-\frac{3\pi }{2},p(t,\xi )=\frac{11}{5},g(t,\xi )=t+\xi ,\sigma (\xi )=\xi ,a=-\pi ,b=-\frac{\pi }{2}.$ It is easy to see that for $t_0>0,$

$$\begin{aligned} \int _{t_0}^{+\infty }\int _{a}^{b}p(s,\xi )[1-\mu (g(s,\xi ))]{\mathrm{d}}\sigma (\xi ){\mathrm{d}}s =\int _{t_0}^{+\infty }\int _{-\pi }^{-\pi /2}\frac{11}{5}\left[ 1-\frac{1}{5}\right] {\mathrm{d}}\xi {\mathrm{d}}s=+\infty . \end{aligned}$$

Then the conditions of Theorem 5 are fulfilled. Therefore every solution of the problem (19), (20) is oscillatory in $(0,\pi )\times [0,\infty )$. Indeed, $u(x,t)=\sin x\cos t$ is such a solution.

### *Example 8*

Consider the partial functional differential equation

21 $$\begin{aligned} & \frac{\partial ^4}{\partial t^4}\bigg [u(x,t)+\frac{1}{2}u(x,t-\pi )\bigg ]=\frac{1}{3}\Delta u(x,t)+ \frac{1}{6}\Delta u\left( x,t-\frac{\pi }{2}\right) \\ & \quad -\int _{-\pi }^{-\pi /2}\frac{1}{6}u(x,t+\xi ){\mathrm{d}}\xi , \quad \ (x,t)\in (0,\pi )\times [0,\infty ), \end{aligned}$$

with the boundary condition

22 $$\begin{aligned} u_x(0,t)+u(0,t)=u_x(\pi ,t)+u(\pi ,t)=0, \quad t\ge 0. \end{aligned}$$

Here $n=4,\mu (t)=\frac{ 1}{ 2},\tau =\pi ,a(t)=\frac{ 1}{ 3},a_1(t)=\frac{ 1}{ 6} ,\rho _1(t)=t-\frac{ \pi }{ 2},p(t,\xi )=\frac{ 1}{ 6},g(t,\xi )=t+\xi ,\sigma (\xi )=\xi ,a=-\pi ,b=-\frac{ \pi }{ 2}.$ It is easy to see that for $t_0>0,$

$$\begin{aligned} \int _{t_0}^{+\infty }\int _{a}^{b}p(s,\xi )[1-\mu (g(s,\xi ))]{\mathrm{d}}\sigma (\xi ){\mathrm{d}}s =\int _{t_0}^{+\infty }\int _{-\pi }^{-\pi /2}\frac{1}{6}\left[ 1-\frac{1}{2}\right] {\mathrm{d}}\xi {\mathrm{d}}s=+\infty , \end{aligned}$$

which shows that the conditions of Theorem 5 are satisfied. By Theorem 5, we obtain that every solution of the problem (21), (22) is oscillatory in $(0,\pi )\times [0,\infty )$. In fact, $u(x,t)=e^{-x}\cos t$ is such a solution.

## Conclusions

This paper provides some oscillation criteria for solutions of higher-order neutral partial functional differential equations with Robin boundary conditions. Using Lemma 1, we obtain Theorems 2 and 4. We should note that Theorem 2 shows that the oscillation of the problem (1), (2) is equivalent to the oscillation of the functional differential equation (4). Using the results in Li and Cui (2001), two useful conclusions are established in Theorems 5 and 6.
